# Awareness of Appendectomy and Its Complications Among Saudis

**DOI:** 10.7759/cureus.46823

**Published:** 2023-10-11

**Authors:** Rehab AlSaleh, Ahmed J Kishta, Abdulrhman A Shamakh, Adnan A Balamesh, Mohamad H Alabaidy, Nawaf A Alsharari, Suleiman I Suleiman

**Affiliations:** 1 Obstetrics and Gynecology, Ibn Sina National College for Medical Studies, Jeddah, SAU; 2 Medicine, Ibn Sina National College for Medical Studies, Jeddah, SAU; 3 Surgery, Ibn Sina National College for Medical Studies, Jeddah, SAU

**Keywords:** open appendectomy, appendicitis, laparoscopic appendectomy, instrumentation, appendectomy

## Abstract

Background: Acute appendicitis is one of the most common surgical causes of acute abdominal pain in adults and children in the emergency department. It is treated by appendectomy by either an open or laparoscopic approach. Although laparoscopic appendectomy has been used for the last 35 years, there are still indications for an open approach in some cases.

Objectives: The aim of this study was to explore the awareness of the general population in Saudi Arabia of appendectomy according to the surgical approach.

Methods: A cross-sectional survey using a questionnaire constructed by an expert based on Google Forms (Google, Mountain View, CA) was used from February to March 2022. Variables were demographical data, general knowledge, history of appendectomy, its surgical approach, and postoperative complications, if any.

Results: The study included 162 participants. The awareness level of acute appendicitis was high (72.2%). History of appendectomy was almost 30% and was significantly more common in males than females (p = 0.045). The rate of postoperative complications showed a significant difference between open (4.3%) vs. laparoscopic approaches (8%) (p = 0.001).

Conclusion: Young, educated Saudis are aware of the importance of surgical intervention for acute appendicitis. However, further hospital-based studies are recommended concerning the role of the surgical approach and its various impacts on postoperative complications.

## Introduction

Acute appendicitis is one of the most common clinical presentations of acute abdominal pain in adults and children in emergency departments [1]. It is routinely treated with surgical dissection of the appendix, an operation known as appendectomy [2], achieved by either an open or laparoscopic approach.

All surgical operations have potential risks that could occur during the surgery or afterward. These complications can be short or long term [3]. A recent study [4] showed that the laparoscopic approach of appendectomy is recommended due to its decreased potentiality of complications. The authors concluded that with laparoscopic surgery, the mortality, morbidity, and hospital stay were significantly reduced in comparison with patients who had open surgery. Nevertheless, differentiation between uncomplicated and complicated appendicitis is essential for the management and prediction of possible complications.

Uncomplicated acute appendicitis is defined as an inflamed appendix without signs of perforation, while complicated appendicitis has necrosis, which eventually may lead to perforation. Moreover, it can be treated by appendectomy, which is the most effective treatment, or by using an antibiotic regimen [5], whereas patients with complicated appendicitis often require surgical intervention [6]. In the past few years, studies showed that appendectomy can be an ambulatory procedure [7-9], although conventional appendectomy still is the mainstay of treatment of acute appendicitis.

Acute appendicitis is a common surgical disease, and the risk of developing it during a lifetime ranges between 7% and 8%. The mortality rate after appendectomy is very low, as it ranges from 0.07% to 0.7% while the postoperative complications rates are much higher, ranging from 10% to 19% in uncomplicated acute appendicitis and reaching up to 30% in complicated acute appendicitis [10].

The cause of acute appendicitis is unknown and still in debate. Acute appendicitis is susceptible to a serious complication, which is obstruction of the appendix lumen, which in turn could cause increased intraluminal pressure with transmural tissue necrosis. Tissue necrosis is followed by the invasion of bacteria, which eventually leads to inflammation of the appendix [11]. A study done by Chen et al. [12] has suggested that delayed appendectomy for acute appendicitis is unsafe due to the risk of increasing hospital stay length and developing postoperative complications. On the other hand, other reports have advocated a conservative attitude toward urgent surgery for acute appendicitis. In the same study, they found that old age (older than 55 years) was the only important risk factor for perforation and one of the predictors for postoperative complications. Also, the study revealed that delayed appendectomy (more than 24 hours) increases the length of hospital stay as a longer delay of appendectomy leads to a longer hospitalization that may lead to increased unnecessary costs for patients [13]. Furthermore, defective diagnosis leads to delayed intervention, which accounts for a high complication rate [12]. Patient factor in late diagnosis is a true dilemma as lack of knowledge or delay in seeking medical treatment is a major contributing factor due to lack of public awareness [13]. Few studies have been conducted to address postoperative complications of appendectomy and we believe that this aspect deserves further research. Thus, this study aims to estimate the level of awareness of appendicitis, the prevalence of appendectomy, and clinical aspects of postoperative complications among the Saudi community.

## Materials and methods

Our study was a cross-sectional study with a non-probability conventional sampling technique. Data were collected from Saudi participants from all over the Kingdom using an online questionnaire. The survey was conducted from February to July 2022, during which 162 responses were recorded.

Sample size calculation was estimated using G*Power software, in which α was 0.05, power was 0.95, and degree of freedom was 5. The minimum sample size was 144. First, sociodemographic and personal characteristics, such as nationality, gender, educational level, and age, were illustrated. Second, participants were asked about their clinical history of diabetes, hypertension, and chronic kidney disease. The following section focused on the assessment of awareness of participants toward acute appendicitis according to questions such as whether they know what appendicitis is, whether they had surgical intervention (appendectomy) or not, if they are aware of clinical symptoms of acute appendicitis or not, how did they seek medical assistance, and why did they seek medical assistance. Questions and responses were scored and awareness was considered if answers to three out of five questions were right (60%). Then, participants were asked what type of surgical intervention they had. Finally, this section was regarding their general condition postoperatively, such as if they had any complications, which were divided into major as abscess and wound infection, and minor as diarrhea, vomiting, and fever. Other complications, such as pain score, duration of hospital stay, and how much time they had until return to their normal activity were noted. Consent was obtained from all participants through the online questionnaire keeping in consideration their information highly confidential. The ethical approval was obtained from Ibn Sina National College for Medical Studies (IRRB-0207052023). Statistical analysis software SPSS version 23 (IBM Corp., Armonk, NY) was used, data were typed on SPSS files and were checked for typing errors. Descriptive statistics were presented as frequency and percentage and comparison was performed using the chi-square test of significance. The level of significance of this study was set at 0.05. The study included all Saudi adults and children above the age of 10 years. The sample was divided into two groups concerning their history of appendectomy. Responses with inaccurate data and non-Saudis were excluded.

## Results

A total of 162 cases participated in the study with a response rate of 100%. The sociodemographic characteristics of Saudi subjects (n = 162) among participants are presented in Table 1. A total of 162 participants were active participants in our study, most of them were between the age of 15-30 years old (83.3%). The majority of subjects were male (52.5%) and females were just under half (47.5%). As for the educational level, university constituted the vast majority of responses (84%), followed by high school (12%).

**Table 1:** Sociodemographic characteristics of Saudi subjects (n = 162) in the Kingdom of Saudi Arabia

Parameter	Number of subjects	Percentage
Gender		
Male	85	52.5%
Female	77	47.5%
Age groups		
0-14 years old	3	1.9%
15-30 years old	135	83.3%
More than 30 years old	24	14.8%
Educational level		
Primary	2	1.2%
Secondary	4	2.5%
High school	20	12.3%
University	136	84%

**Figure 1:**
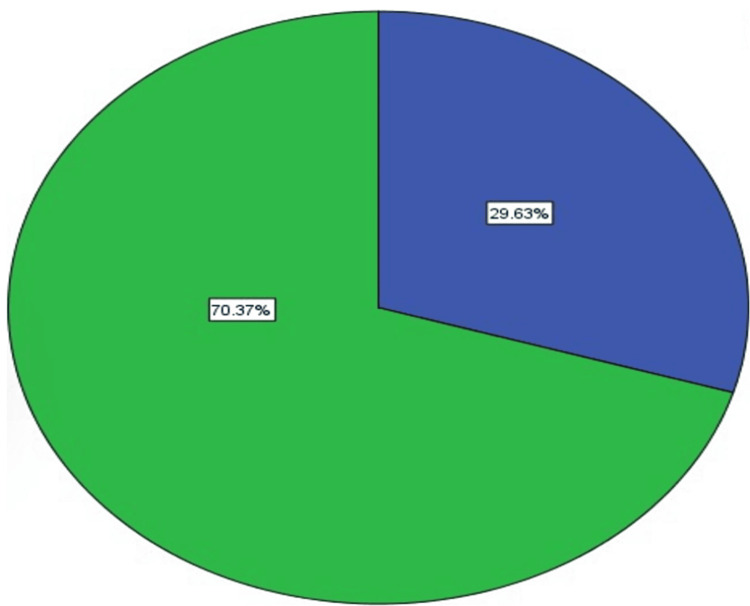
Distribution of the study subjects according to having appendix removed or not Yes is represented in blue color and no is represented in green color.

Figure 1 shows the percentage of participants who had been exposed to appendectomy. Overall, the majority of participants did not have their appendix removed (70%), whereas the remaining participants (almost 30%) had their appendix removed.

Table 2 presents the awareness of appendicitis among Saudis. Among the respondents, the majority (72.20%) were knowledgeable about appendicitis according to the criteria selected by the study group.

**Table 2:** Awareness of appendicitis among Saudi subjects (n = 162) in the Kingdom of Saudi Arabia

Parameter	Number of subjects	Percentage
Awareness of appendicitis		
Aware	117	72.2%
Not aware	45	27.8%

Table 3 shows the demographic characteristics of cases that had an appendectomy. There was a significant statistical difference between males and females. Appendectomy was more common among males (p = 0.045). On the contrary, age was not found to be a significant factor in differentiation between both groups (p = 0.361).

**Table 3:** Demographic characteristics of cases with appendectomy in comparison to cases without appendectomy

Parameters	Categories	Number of cases with appendicectomy (%)	Number of cases without appendectomy (%)	P-value
Gender	Male	31 (19.1)	54 (33.3)	0.045
	Female	17 (10.5)	60 (37)	
Age groups	0-14	2 (1.2)	1 (0.6)	0.361
	15-30	39 (24.1)	97 (59.9)	
	More than 30	7 (4.3)	16 (9.9)	

Table 4 shows the rate of postoperative complications according to the type of surgical approach. The type of operation that was significantly associated with postoperative complications was the laparoscopic approach (p = 0.001).

**Table 4:** Factors associated with postoperative complications of appendectomy among Saudi subjects (n = 162) in the Kingdom of Saudi Arabia

Parameters	Categories	Number of cases with postoperative complication (%)	Number of cases without postoperative complication (%)	P-value
Type of operation	Open appendectomy	7 (4.3)	16 (9.9)	0.001
	Laparoscopic appendectomy	13 (8)	12 (7.4)	

Table 5 shows the rate of postoperative complications according to the type of surgical approach. The type of operation that was significantly associated with postoperative complications was the laparoscopic approach.

**Table 5:** Type of surgical approach in relation to complications

Type of operation	Fever	Diarrhea	Vomiting	Antibiotic-related rash	Constipation	Abscess	Wound infection	
Laparoscopic	5	0	2	1	2	1	8	
Open	1	1	1	0	2	3	2	
Total	6	1	3	1	4	4	10	

## Discussion

The current study aimed to evaluate the awareness of the Saudi community in relation to acute appendicitis. It is managed mainly surgically; however, surgical operations have potential complications that could occur during the operation or post surgery. These complications can be short or long term, depending on several variables such as sociodemographic factors and mainly the type of surgical approach [3]. This shows the importance of measuring the public awareness of appendicitis and its complications [14]. The cost of which type of operation to be chosen has a remarkable influence on measuring the outcome of each surgical approach [15]. In terms of gender, a study conducted by two research groups [16,17] found that the incidence of acute appendicitis in males and females followed a similar pattern but males had higher rates at all ages than females, which is in line with what we found in our study. On the other hand, a study conducted by Salö et al. [18] stated some variability in terms of gender, as postoperative complications were noticed more often in females than males. The highest incidence of appendicitis according to age group in our study was in the third decade. However, in another study, they found that the vast majority of patients with appendicitis were in the second decade [16]. We believe this conflict might be related to the difference in sample size and inclusion and exclusion criteria. The majority of our sample had a high level of awareness of appendicitis, which is compatible with what other researchers found [19,20]. Even though, those studies were conducted in various continents, the global insight of appendicitis is obvious. The number of cases with laparoscopic and open appendectomy groups was almost similar. Both groups complained of postoperative pain in the first two weeks and roughly less than a quarter of cases with the laparoscopic approach were pain-free for the same phase, as the severity of tissue disruption was limited [21]. However, both groups behaved almost the same afterward [22]. It is well known that indications for the open approach are totally different than the laparoscopic approach, which will categorize them into two different groups.

Many studies suggested that the laparoscopic approach is safe and convenient, and leads to less incidence of postoperative complications and shorter duration of postoperative stay in hospital and earlier return to normal activity [23-30]. However, in this study, postoperative fever and wound infection were higher in the laparoscopic approach. These findings are not consistent with modern literature and that might suggest the need to improve infection control techniques. Moreover, the increased incidence of abscesses in the open appendectomy group is a defective outcome, and that might be due to the nature of the affected cases.

Limitations of this study were the study design of non-randomization, which made generalization of the results limited. Another limitation was the postoperative complications were taken from participants' responses, not hospital records.

## Conclusions

In conclusion, our study found that the general level of awareness about acute appendicitis is good in young educated Saudis. However, larger randomized community studies are recommended to explore the extent of awareness among the general population. This will have an impact on reducing the number of cases that may arrive at the hospital in advanced stages.
